# Prevalence and determinants of unintended pregnancy among pregnant woman attending ANC at Gelemso General Hospital, Oromiya Region, East Ethiopia: a facility based cross-sectional study

**DOI:** 10.1186/s12905-016-0335-1

**Published:** 2016-08-17

**Authors:** Faiza Mohammed, Abdulbasit Musa, Abdella Amano

**Affiliations:** 1 Head of Obstetrics ward midwives, Gelemso General Hospital, Oromiya region, Gelemso, Ethiopia; 2 Departments of Midwifery, College of Health and Medical Sciences, Haramaya University, Harar, Ethiopia; 3 Department of biostatistics and epidemiology, school of public and Environmental Health, Hawassa University, Hawassa, Ethiopia

**Keywords:** Prevalence, Determinants, Unintended pregnancy, East Ethiopia

## Abstract

**Background:**

Unintended pregnancy is among the major public health problems that predispose women to maternal death and illness mainly through unsafe abortion and poor maternity care. The level of unintended pregnancy is high in developing countries. Hence, the purpose of this study is to assess the prevalence of unintended pregnancy and the associated factors among pregnant woman attending antenatal care at Gelemso General Hospital, East Ethiopia.

**Methods:**

A facility-based cross-sectional study was conducted from January 10 to April 13, 2015 among women who had attended antenatal care at Gelemso General Hospital. A systematic random sampling technique was used to select a sample of 413 participants. Data were collected via face-to-face interview using a structured and pre-tested questionnaire. Bivariate and multivariate analyses were made to check the associations among the variables and to control the confounding factors.

**Results:**

Out of the 413 pregnancies, 112 (27.1 %) were unintended of which 90(21.9 %) were mistimed, and 22(5.2 %) were unwanted. Multivariate analysis revealed that single, divorced/widowed marital statuses, having more than 2 children, and having no awareness of contraception were significantly associated with unintended pregnancy.

**Conclusion:**

Over a quarter of women had an unintended pregnancy, a rate which is lower than previously reported. Designing and implementing strategies that address contraceptive needs of unmarried, divorced and widowed women, creating awareness of contraceptives at community level and reinforcing postnatal contraceptive counseling to all mothers giving birth at health institution is recommended to reduce the rate of the unintended pregnancy among parous women.

**Electronic supplementary material:**

The online version of this article (doi:10.1186/s12905-016-0335-1) contains supplementary material, which is available to authorized users.

## Background

Unintended pregnancy is a pregnancy that is either unplanned or unwanted at the time of conception, and it is a significant public health concern in the world nowadays [1]. Analysis of Demographic and Health Survey (DHS) data shows that the magnitude of unintended pregnancy in developing countries ranges from 14 % to 62 % of all births. The highest rate of unintended pregnancy occurs in Sub-Saharan Africa, where about 86 unintended pregnancies occur for every 1000 women of reproductive age [2].

Unintended pregnancy is an important public health problem that predisposes women to maternal deaths and illnesses mainly through unsafe abortions and poor maternity care. It is associated with late initiation and inadequate utilization of antenatal care services [3–6], maternal depression and anxiety [7–9] and smoking and drinking behaviors during pregnancy [5–10].

Of the estimated 210 million pregnancies that occur globally every year, about 80 million (40 %) are unintended (mistimed and/or unwanted) [11] and one in ten of these pregnancies end in unsafe abortions [12] and around 14 million abortions are from sub-Saharan Africa [11].

Family planning is one of the most effective strategies in reducing maternal death due to unwanted pregnancy and risks of unsafe abortion. It can also prevent closely spaced and ill-timed pregnancies and births, which contribute to high infant mortality rate in developing world [13].

Having high unmet need for family planning (25 %) and low Antenatal Care(ANC) coverage(34 %), Ethiopia is one of the countries with highest maternal (676 death/100,000 live birth) and child mortality rate (88/1000 live birth) in the world [14]. Cognizant of this, the Ethiopian government prepared national reproductive health strategy that gave stress on the importance of reducing unintended pregnancy through raising the contraceptive use to 66 % [15],which otherwise leads to an estimated 382,000 induced abortion per year [16].

In addition, ranges of reproductive health services offered for women of reproductive age group including family planning services, education, counseling and assessment of sexual reproductive need, education and counseling on Human immune deficiency Virus and other Sexually transmitted infection, ANC, skilled delivery, basic and comprehensive emergency obstetric and new born care, and comprehensive abortion care were declared to be free for all people regardless of their ability to pay [17].

Even if family planning services are available free of charge, unwanted pregnancy is still one of the remaining challenges in reducing maternal mortality in Ethiopia. According 2011 Ethiopian Demographic and Health Survey (EDHS), 25 % of the women with births in the five years before the survey and 32 % of the current pregnancies were reported to be unintended [14].

Previous Studies conducted in different countries showed wide ranges of correlates of unintended pregnancy. Study from Kenya showed the association between unintended pregnancy with marital status, Employment status, ethnicity and residence areas [18]. The analysis of Nepali DHS revealed the association between unintended pregnancy and age of the women, religion, exposure to media, and knowledge of contraceptives [19]. In Ethiopia, significant association was found between unintended pregnancy and high parity, longer estimate time to walk to nearest health care facility, economic status of the women [13]. Majority of Ethiopian studies on unintended pregnancy have relied largely on national large-scale data. Little is therefore known about the prevalence and determinants of unintended pregnancy at sub-national level. Hence, this study aimed to assess the prevalence of unintended pregnancy and its associated factors among women who gave birth at Gelemso General Hospital, Ethiopia.

## Methods

This study was conducted at Gelemso General Hospital, which is found in Oromiya National Regional State, and located 376 kms from Addis Ababa. The hospital has a catchment population of 1,149,106, with 142 beds distributed in medical, pediatrics, surgical, gynecology, and obstetrics wards. Monthly, an estimated of 432 clients attend the antenatal clinic. The institution based cross-sectional study was conducted from January 10 to April 13, 2015.

The sample size was determined using single proportion formula (*n* = Zα/2^2^p (1-p)/d^2^) by considering the following assumptions; proportion of unintended pregnancy of 50 % (an estimate only as no previous data were available), 95 % level of confidence (Z = 1.96), 5 % of marginal error (d = 0.05), and 10 % of non response rate making final sample size of 422.

A systematic random sampling method was used after case review of previous three months in the hospital had been identified, and found to be 1296. Hence, every third women (K = 1296/422) was selected to be included in the study.

Data on socio-demographic information (age, marital status, residence, maternal occupation, maternal and partner’s educational status, average monthly income, availability of radio or TV) and obstetric factors (Parity, birth interval, history of abortion, previous history of unintended pregnancy, pregnancy status, history of contraceptive use, final decision maker) were collected using a pre-tested and structured questionnaire. After pretesting, sensitivity analysis for the questionnaire was done and the tool was found to be reliable with cronbach’s alpha (r) of 0.87. The questionnaire is available to be read as Additional file 1. Data were collected through face-to-face interviews after the data collectors and the supervisors had been trained for the purpose. The data were collected from the respondents after they had received the ANC service, and the interview took a maximum of 30 minutes. And in order to get genuine responses from the respondents, female data collectors were used.

A participant’s intention of last pregnancy was obtained by inquiring her whether she conceived at the exact time she wanted pregnancy, whether she wanted to delay the pregnancy for sometime (mistimed), and whether she didn’t want the pregnancy at all (unwanted). Furthermore, an unintended pregnancy was identified as either mistimed or unwanted. Parity in our study is defined as having birth after 28 weeks of gestation regardless of birth outcome (still birth or alive). Moreover, the final decision maker on the study participant’s health is defined as the one who has the final power to give decision on whether the woman can receive health care or not.

exported to SPSS Version 20.0 software package for analysis. The results were presented in tables and text using frequency and summary statistics such as mean, standard deviation, and percentage. The data were analyzed using logistic regression to determine the effect of various factors on the outcome variable and to control confounding.

Most of the variables were fitted to the bivariate logistic regression. Then all the variables with *p* ≤ 0.2 in the bivariate analysis were further entered into the multivariate logistic regression model. In the multivariate analysis, standard enter techniques were fitted. The variables having *p* value ≤ 0.05 in the multivariate analysis were taken as significant predictors. Crude and adjusted odds ratios with their 95 % confidence intervals were calculated. The Hosmer and Lemeshow goodness of- fit test was used to assess whether the necessary assumptions for the application of multiple logistic regression were fulfilled, and the *p* value > 0.05 was considered a good fit.

Ethical clearance was obtained from the Institutional Research Ethics Review of Haramaya University, College of Health and Medical Sciences, and a formal letter of cooperation was written to west Hararge Health Office and Gelemso General Hospital. After explaining the purpose of the study, the data collectors had obtained voluntary verbal consent from each study participant. The participants were informed that participation was on voluntary basis and they could withdraw at any time if they were not comfortable about the questionnaire. Personal identifiers were not included so that a participant’s confidentiality was assured.

## Result

### Scio-demographic characteristics of the respondents

Of the 422 sampled women, 413 were included in the analysis giving a response rate of 97.9 %. Their mean age was 25.25 years (SD ± 4.7 years) and 327(79.2 %) of them were in age ranges of 18-34 years. Majority, 238(57.6 %) of the women reside in rural area. Three hundred and seventy (89.6 %) of them were married, 322(78.0 %) were Muslim by religion. Three hundred and thirty (79.9 %) of them was Oromo by ethnicity and 299 (72.4 %) had no occupation (house wife). Three-quarter of the respondents (74.8 %) and 252(61.0 %) of their partners did not attend formal education. Majority of the women, 302(73.1 %), have radio/TV at their home. Two hundred and forty (58.1 %) of them have access to health service within 10 km (Table 1).

**Table 1:** Socio-demographic characteristics of the women attending ANC at Gelemso general hospital (*n* = 413), Oromiya region, East Ethiopia, January 10 to April 13, 2015

Variables	Frequency (Number (%))
Maternal age	
< 18 years	41(9.9)
18–34	327(79.2)
≥ 35 years	45(10.9)
Place of Residence	
Urban	175(42.4)
Rural	238(57.6)
Marital Status	
Married	370(89.6)
Single	27(6.5)
Divorced/widowed	16(3.9)
Religion	
Muslim	322(78.0)
Christian	91(22.0)
Ethnicity	
Oromo	330(79.9)
Amhara	62(15)
Gurage/Tigrea	21(5.1)
Occupation	
Housewife	299(72.4)
Government Employee	86(20.8)
Private worker	28(6.8)
Maternal Education	
No formal education	309(74.8)
Primary education	49(11.9)
Secondary education	43(10.4)
Tertiary education	12(2.9)
Partner’s Education	
No formal Education	252(61.0)
Primary Education	64(15.5)
Secondary Education	81(19.6)
Tertiary Education	16(3.9)
Have media source at their home	
Yes	302(73.1)
No	111(26.9)
Distance from nearby health facility	
< 10 km	240(58.1)
> 10 km	173(41.9)

### Obstetric characteristics of the respondents

Of all the participants, 264 (63.9 %) were multiparous. Before 18 years of age, 169(40.9 %) of the women were married and 117(28.3 %) had given birth to their first child. Most of them (87.2 %) desired a birth- interval of > 2 years, but 231(87.5 %) of the multiparous respondents had already had a birth interval of two and above years. More than half of the respondents 219(53.0 %), wanted to have six or more children, and 134 (32.4 %) of these reported to have previous history of abortion. Two hundred and fourteen of the participants (51.8 %) have ANC follow up before their last visit, with a mean number of ANC visits of 2.7 (SD ± 0.6).

**Fig. 1:**
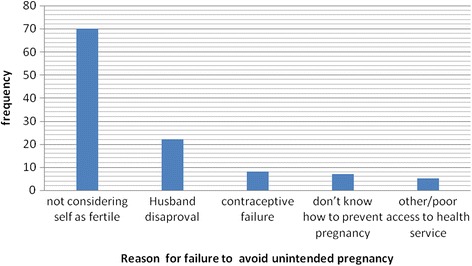
Reasons given for unintended pregnancy, among mother with unintended pregnancy (*n* = 112), Gelemso General Hospital, Oromiya region, East Ethiopia, January 10 to April 13, 2015

Regarding the current pregnancy status, 112(27.1 %) of the pregnancies were unintended of which 90(21.9 %) were mistimed and 22(5.2 %) unwanted. One woman out of ten (11.6 %) reported to have experienced unwanted pregnancy. Almost all of the respondents (95.9 %) heard about contraceptive (Table 2). Of those who reported to have unintended pregnancy, 70(62.5 %) said that they had unintended pregnancy because they believed that they were not fertile (Fig. 1).

**Table 2:** Obstetric characteristics of women attending ANC at Gelemso general hospital (*n* = 413), Oromiya region, East Ethiopia, January 10 to April 13, 2015

Variables	Frequency (Number (%)
Parity	
primi	149(36.1)
1–2	100(24.2)
3–5	109(26.4)
above 5	55(13.3)
Age at first marriage	
less than 18 years	169(40.9)
18 years and above	244(59.1)
Age at first pregnancy	
less than 20 years	117(28.3)
20 years and Above	296(71.7)
Wanted birth interval	
< 2 years	53(12.8)
> 2 years	360(87.2)
Desired number of children	
1–2	22(5.3)
3–5	172(41.6)
6 and above	219(53.0)
Interval between the last two delivery(*n* = 264)	
< 2 years	33(12.5)
> 2 years	231(87.5)
Presence prior of ANC follow-up	
Yes	214(51.8)
No	199(48.2)
History of abortion	
Yes	134(32.4)
No	279(67.6)
Current pregnancy status	
Intended	301(72.9)
Un Intended	112(27.1)
Previous history of unwanted pregnancy	
yes	48(11.6)
No	365(88.4)
Heard about contraceptives	
Yes	396(95.9)
No	17(4.1)
Decision maker about respondent’s health care	
Me only	50(12.1)
Me and my husband	339(82.1)
My husband only	24(5.8)

### Determinants of unintended pregnancy among the respondents

In the bivariate analysis, the factors that were significantly associated with unintended pregnancy were marital status, age of the mother, parity and having heard about contraceptives. From these variables, marital status, parity, and awareness on contraceptive were significantly and independently associated with unintended pregnancy in multiple logistic regression analysis.

The single respondents were 5 times (AOR = 5.5, 95 % CI = 2.25, 13.64) and the divorced/widowed respondents were 4 times (AOR = 4.0, 95 % CI = 1.31, 12.45) more likely to have unintended pregnancy than the married ones.

The mothers with parity of 3–5 (AOR = 2.37, 95 % CI = 1.36, 4.15), and with that of >5 (AOR = 4.76, 95 % CI = 2.4, 9.65) were 2.4 and 4.8 times more likely to experience unintended pregnancy compared to those who had two or fewer children. Women who didn’t hear about contraceptives were 2.7 times (AOR = 2.73, 95 % CI = 1.15, 6.50) more likely to have unintended pregnancy compared to those who had heard about contraceptives (Table 3).

**Table 3:** Bivariate and Multivariate analysis of factors associated with unintended pregnancy among women attending ANC at Gelemso Hospital, Oromiya region, East Ethiopia, January 10 to April 13, 2015

Variable	Pregnancy status		COR(95 % CI)	AOR(95 % CI)
	Unintended	Intended		
	Number (%)	Number (%)		
Marital status				
Married	87(23.5)	283(76.5)	1	
Single	11(40.7)	16(59.3)	4.7(2.12, 10.58)	5.5(2.25, 13.64) *
Divorced/widowed	7(43.8)	9(56.2)	4.18(1.51, 11.56)	4.0 (1.31, 12.45) *
Age of the women in year				
Less than 18 years	18(43.9)	23(56.1)	1	
18–34	77(23.5)	250(76.5)	0.39(0.20,0.77)	0.51(0.23, 1.14)
35 and above	17(37.8)	28(62.2)	0.78(0.33,1.84)	0.58(0.20,1.63)
Parity				
< 2	51(20.5)	198(79.5)	1	
3–5	36(33.0)	73(67.0)	1.92(1.16,3.17)	2.37(1.36, 4.15) *
Above 5	25(45.5)	30(54.5)	3.2(1.75, 5.98)	4.76(2.40, 9.65) *
History of unwanted pregnancy				
Yes	19(39.6)	29(60.4)	1.92(1.03, 3.58)	1.86(0.94, 3.66)
No	93(25.5)	272(74.5)	1	
Heard about contraceptives				
Yes	97(25.3)	287(74.7)	1	
No	15(51.7)	14(48.3)	3.17(1.48, 6.81)	2.73(1.15, 6.50) *

*Significant at *p*-value of ≤ 0.05

## Discussion

Unintended pregnancy is an important public health problem that predisposes women to maternal deaths and illnesses mainly through unsafe abortions and poor maternity care. It is associated with late initiation and inadequate utilization of antenatal care services [3–6], maternal depression and anxiety [7–9] and smoking and drinking behaviors during pregnancy [5–10].

In this study unintended pregnancy was 27.1 % among the study participants. In this study unintended pregnancy was 27.1 % among the study participants. This finding is in agreement with the finding from other studies in Ethiopia [20, 21] and Kenya [18] but it is lower than the other studies that were conducted in Jimma 35 % [22] and Hosanna (34 %) [19]. This might be due to the increased availability and accessibility of maternal health services, including access to modern contraceptives with time since that time. Moreover, this finding is lower than the findings of studies conducted in Tanzania (45.9 %) [23] and Nepal (41 %) [19]. This might be due to the socio-cultural and health coverage differences among the countries.

In this study, considering oneself as not fertile, 70(62.5 %) is the most common reason mentioned by respondents for unintended pregnancy. A similar finding is reported from a study in Ethiopia [24] and other studies conducted in China [25] and Indonesia [26]. This shows the existence persistence gaps in creating awareness of reproductive aged women regarding appropriate usage of contraceptives. In-depth investigation on such women is advised to assess the root reasons for failure to considering oneself as fertile that can help in developing evidence based intervention.

In this study marital status was significantly associated with unintended pregnancy. The women who were single and divorced/widowed were more at a risk of unintended pregnancy than the married ones. This finding was consistent with the ones reported from Tanzania [23] and Kenya [18] which might be due to the fact that single and divorced/widowed women were more likely to be involved in sexual activities for motivations such as pleasure, social status, or other exchanges than for childbearing. The other possible reason might be the fact that women with extra marital union were less likely to get access to contraceptives, as they feel ashamed of their sexual activities, as evidenced by another Ethiopian study [27].

In this study, parity was also significantly associated with unintended pregnancy. Parity 3 and above women were more likely to experience unintended pregnancy than those who were < 2 parity. This finding is consistent with a finding of other studies in Ethiopia [20], which showed an increased risk of unintended pregnancy with increased parity. This is due to the fact that high parity woman might already have adequate children and practice sex for enjoyment rather than to have children. In addition, it might imply the gaps in counseling and provision of postpartum contraceptives.

Awareness of contraceptive was significantly associated with unintended pregnancy. Those participants who never heard about contraceptive were more likely to experience unwanted pregnancy. This finding is also consistent with findings in Ethiopia [21, 28] and other countries [19, 22]. This implies the importance of raising the awareness of the women on contraceptive, as an important strategy in tackling unintended pregnancy and its consequences.

### Limitations

Since the study is a facility based, it might not indicate the true rate of unintended pregnancy in the community, as many of the clients with unintended pregnancy had less chance to visit the maternal health care service, including ANC [22]. It is also difficult to establish a temporal relationship as the study design was cross-sectional, and the wider confidence interval observed with some variables may also indicate inadequate sample size. Furthermore, as this study focuses particularly on unintended pregnancy that ended with child birth, finding might not be generalizable to unintended pregnancy that ended with abortion. Despite these limitations, the finding of this study is expected to contribute a lot to the understanding of the factors associated with unintended pregnancy in the study area.

## Conclusion

Over a quarter of women had an unintended pregnancy, a rate which is lower than previously reported. Marital status, Parity and having information about contraception were among the variables that have significant association with unintended pregnancy. Designing and implementing strategies that address contraceptive needs of unmarried women, creating awareness on contraceptives at community level and reinforcing postnatal counseling regarding contraceptive to all mothers giving birth at health institution were recommended in order to reduce the rate of the pregnancy among parous women. In addition, future research should address the determinants of unintended pregnancy at earlier pregnancy stage to address all the possible pregnancies that end with abortion or live birth.

## Abbreviations

ANC, antenatal care; DHS, demographic and health survey; EDHS, Ethiopian demographic and health survey

## Additional file

Additional file 1: Part One: Socio-demographic characteristics of the respondent. (DOCX 20 kb)
