# Supplementary material for: Prevalence and determinants of unintended pregnancy among pregnant woman attending ANC at Gelemso General Hospital, Oromiya Region, East Ethiopia: a facility based cross-sectional study
Source: BMC Womens Health. 2016 Aug 17;16:56. doi: 10.1186/s12905-016-0335-1 (PMC4989486; doi:10.1186/s12905-016-0335-1)
Supplement: Additional file 1: — Part One: Socio-demographic characteristics of the respondent. (DOCX 20 kb) [file 12905_2016_335_MOESM1_ESM.docx]

| Part One: Socio-demographic characteristics of the respondent | | | |
| --- | --- | --- | --- |
| Serial no | Questions | Choice answer | Skip to |
| 101 | Age of the mother | In year-------- |  |
|  | place of residence | 1. Rural 2. Urban |  |
| 102 | Marital status | 1.Married 2.Divorced  3.Widowed 4.Never married  5.Separated |  |
| 103 | Religion | 1.Muslim 2.Orthodox  3,Protestant 4,Others specify-------- |  |
| 104 | Ethnicity | 1. Oromo 2. Amhara 3. Somali 4. Gurage  5..Other ,specify-------- |  |
| 105 | Occupation | 1. House wife 2.Gov’t Employee  3. Farmer 4. Private work  5. Daily laborer 6. Student  7.Others specify--- |  |
| 106 | Maternal education | 1.Not able to write and read  2.able to write and read  3.Primary education  4.Secondry education (9-12 )  5. college or university |  |
| 107 | Paternal educational Status. | 1.Not able to write and read  2.able to write and read  3.Primary education  4.Secondry education(9-12 )  5.college or university |  |
| 108 | Average monthly family income. | In birr--------------------- |  |
| 109 | Do you have any of the following means of communication at your home? | 1. Radio 2. TV  3. none 4.others, specify---------- |  |
| 110 | How long it takes you to reach your nearby health center/hospital in minutes? | In minutes-------------- |  |
| 111 | How long it takes you to reach your nearby health center/hospital in kilometer? | In kilometer-------------- |  |

| Part two: Obstetric factors of the respondents | | | |
| --- | --- | --- | --- |
| S.No | Question | Choice Answer | skip |
| 201 | What is your age when you get married? |  |  |
| 202 | At what age you conceived your first child? |  |  |
| 203 | How many births have you ever had (parity)? | In number-------------------------------- |  |
| 204 | what is the interval between your last birth and the one preceding this one?(only asked if she is not primi,) | in number------------- |  |
| 205 | In what interval you want to deliver your children( in year) | in year------------------ |  |
| 206 | How many children you want to have in your life? | in number-------------- |  |
| 207 | Have you ever had abortion? | 1. Yes 2. No |  |
| 208 | Did you have prior antenatal visit during your this pregnancy? | 1.Yes  2. No | 210 |
| 209 | How many times you visited health facility for this pregnancy? | In number-------------------------------- |  |
| 210 | Have you got pregnant at exact time you want to conceive? | 1. Yes  2. No |  |
| 211 | If ‘No’ for Q210, did you want to delay your pregnancy for some times? | 1. Yes 2. No | 214 |
| 212 | If ‘No’ for Q211, didn’t you want to conceive at all | 1. Yes 2. No |  |
| 213 | If your pregnancy is not wanted or mistimed, why you get pregnant? | 1. My husband wanted it 2. I conceived incidencially while using contraceptives 3. I haven’t consider my self as fertile while I got pregnant 4. I didn’t know how to prevent pregnancy |  |
| 214 | Did you have previous history of un intended pregnancy? | 1. Yes 2. No |  |
| 215 | Have you ever heard about contraceptives? | 1. Yes 2. No |  |
| 216 | Which types of family planning you have heard? | 1. OCP 2. Injectable 3. Implants 4. IUCD 5. Other, specify |  |
| 217 | Have you ever used any contraceptives? | 1. Yes 2. No |  |
| 218 | If yes, which types of contraceptives | 1. OCP 2. Injectable 3. Implants 4. IUCD 5. Other, specify |  |
| 219 | Who is the final decision maker on your health care? | 1. My husband alone 2. Me alone 3. Both my husband and me 4. Other, specify |  |

***Thank you!!!***
